# Endovascular treatment of intracranial vertebral artery unruptured dissecting aneurysms: Comparison of flow diversion and stent-assisted coiling or stenting alone

**DOI:** 10.3389/fneur.2022.919866

**Published:** 2022-08-23

**Authors:** Li Li, Gang-Qin Xu, Hui-Li Gao, Bu-Lang Gao, Kun Zhang, Zi-Liang Wang, Tian-Xiao Li

**Affiliations:** Henan Provincial People's Hospital, Zhengzhou University, Zhengzhou, China

**Keywords:** intracranial aneurysms, dissecting, vertebral artery, flow diverter, effect

## Abstract

**Purpose:**

To investigate the effect and safety of flow diverters in the treatment of unruptured dissecting intracranial aneurysms of the vertebral artery in comparison with stent-assisted coiling or stenting alone.

**Materials and methods:**

Patients with unruptured dissecting intracranial aneurysms of the vertebral artery treated with the flow diverter, stent-assisted coiling, or stenting alone were retrospectively enrolled. The clinical data were analyzed and compared.

**Results:**

Twenty-five patients were enrolled in the flow diversion group and 42 patients in the stenting group. Twenty-six flow diverters were deployed in the flow diversion group. Immediate angiography revealed contrast agent retention within the aneurysm cavity in all patients. In the stenting group, 48 stents were deployed, and immediate angiographic outcome showed O'Kelly-Marotta (OKM) grade D in 18 (42.9%) aneurysms, grade C in 16 (38.1%), and grade B in 8 (19.0%). Periprocedural ischemic complications of thrombosis occurred in two (4.8%) patients and were treated with thrombolysis. In the flow diversion group, 19 (76%) patients underwent angiographic follow-up 3–46 (median 24) months after the procedure, with the OKM grade D in 11 (57.9%) patients, C in two (10.5%), and B in six (31.6%). The aneurysm recurrence rate was zero, and all diverters remained patent. Asymptomatic instent stenosis occurred in two (10.5%) patients. In seven of the ten patients with mild or moderate parent artery stenosis before the procedure who experienced angiographic follow-up, the stenosis was improved in five (71.4%) patients. In the stenting group, angiographic follow-up was carried out in 33 (78.6%) patients 6–58 months (median 34) after the procedure, with OKM grade D in 22 (66.7%) patients, grade C in five (15.2%), grade B in three (9.1%), and aneurysm recurrence (grade B, with increased contrast agent into the aneurysm cavity) in three (9.1%). Five (16.7%) patients experienced asymptomatic instent stenosis, and six of the 12 patients (50%) with parent artery stenosis were improved.

**Conclusion:**

Flow diverters with or without selective adjunctive coiling for the treatment of unruptured dissecting intracranial aneurysms of the vertebral artery may be safe and effective with good occlusion effects not inferior to those of stent-assisted coiling and stenting alone even though the long-term effect still warrants confirmation.

## Introduction

As a cause of stroke, spontaneous dissection of the intracranial vertebral artery may present with varied symptoms in young adults like ischemic symptoms, subarachnoid hemorrhage, brainstem compression, and local symptoms (headache), and has been increasingly recognized with improvement of imaging technology (1, 2). Arterial dissection is probably caused by a disruption of arterial internal elastic lamina and media, resulting in penetration of flowing blood into the arterial wall and formation of an intramural hematoma (3, 4). Arterial dissection may exhibit different shapes like dilatation, stenosis, or both based on the tear depth, with luminal stenosis or occlusion caused by subintimal dissection and luminal dilatation by subadventitial dissection (dissecting aneurysm). Dissecting aneurysms on the intracranial segment of the vertebral artery occur mostly in male young adults and are the most important reason for subarachnoid hemorrhage and posterior circulation stroke (1). Currently, there are no established treatment approaches for unruptured dissecting aneurysms of the vertebral artery. Even if unruptured intracranial dissecting aneurysms of the vertebral artery have been thought to have a benign clinical course (2, 5–10), the natural course of these dissections are still unknown and treatment guidelines remain controversial and debatable (5, 11, 12). Moreover, some researchers believed that the risk of bleeding from unruptured dissecting aneurysms of the vertebral artery was higher than previous reports (6, 12). Patients with these dissecting aneurysms may experience serious neurological deficits after hemorrhage and secondary ischemic events, and early management of these aneurysms is necessary because rebleeding after rupture is common (2, 8, 9). However, intracranial dissecting aneurysms of the vertebral artery are usually wide-necked and involve a long segment and the posterior inferior cerebellar artery, which makes it difficult for traditional treatment approaches. Anticoagulation is preferred for conservative treatment of hemodynamically stable dissecting aneurysms (13), however, use of anticoagulation or antiplatelet therapy in patients with ischemic presentations of unruptured vertebral arterial dissections may exacerbate aneurysmal dissection and cause a rupture of the dissecting aneurysm (12). One randomized trial had demonstrated no difference in the effect of antiplatelet and anticoagulant therapy at preventing stroke and death in patients with symptomatic vertebral and carotid artery dissections (14). A deconstructive approach is to surgically or endovascularly occlude the dissecting segment, but some disadvantages may be involved, including ischemic events in the case of the dominant vertebral artery being occluded, and the need of a bypass in the case of involvement of the posterior inferior cerebellar artery by the dissecting aneurysm (12). As a reconstructive technique, stent placement and stent-assisted coiling have been applied to treat intracranial vertebral artery dissecting aneurysms, with good outcomes being achieved (15–17). Flow diverting devices are used to reconstruct the parent artery of the aneurysm and have achieved good prognoses in the treatment of complex anterior circulation aneurysms, but their application in the posterior circulation is still in the exploratory stage. It was hypothesized that the flow diverters could be safely applied to effectively treat the intracranial dissecting aneurysms of the vertebral artery. This study was consequently conducted to investigate the safety and effect of flow diverters in the treatment of this condition in comparison with stent placement or stent-assisted coiling.

## Materials and methods

This retrospective case-control single-center study was approved by the ethics committee of our hospital, with the written informed consent obtained from all patients to participate. Patients with unruptured dissecting intracranial aneurysms of the vertebral artery who were treated with stent placement, stent-assisted coiling or flow diverters were enrolled between May 2014 and October 2019. The inclusion criteria were patients with imaging-confirmed unruptured dissecting intracranial aneurysms on the intracranial segment of the vertebral artery, with increased dissecting size on repeated angiographic imaging, and sustained contrast stagnation in the dissecting aneurysms, which were treated with stent placement, stent-assisted coiling or flow diverters. The exclusion criteria were patients with infectious or traumatic intracranial aneurysms, non-dissecting aneurysms, history of endovascular or surgical treatment, and concomitant with intracranial tumors or other diseases affecting management of intracranial aneurysms. Patients were divided into the stent group to receive stent-assisted coiling or stent placement alone and the flow diversion group who were to receive flow diverting treatment. Generally, in some complex lesions, in which there was no clear aneurysm sac to effectively fill the coil, the lesion segment was long, and the dissection shown by on magnetic resonance imaging was larger than that shown on digital subtraction angiography, the Pipeline device would be considered as the first choice even though ordinary stents might also be selected because of their low price.

Before the endovascular embolization procedure, patients were given dual antiplatelet therapy (aspirin 100 mg/d and clopidogrel 75 mg/d), administered orally for 3–5 days. Thromboelastography (TEG) was conducted 3 days after the administration of the therapy, and the dose was adjusted according to the TEG test results so as to maintain the inhibition rate of arachidonic acid (AA) > 50%, the inhibitive rate of adenosine diphosphate (ADP) > 30%, and the maximal amplitude of ADP curve at 31–47 mm. Intravenous bolus injection of heparin 50–70 U / kg was administered before the endovascular treatment, and heparin was then maintained at 1,000 U / h. After the embolization, dual antiplatelet therapy was continued at the same dosage for 6 months before switching to aspirin at the same dosage for at least 1 year.

The endovascular procedure was performed under general anesthesia. After a 6F long sheath was inserted into the right femoral artery, a 6F Navien intermediate catheter (Medtronic, USA) was navigated to the aneurysm. The stent delivery system was sent over a micro-guidewire to the appropriate location for deployment of a stent (Neuroform stent, Stryker, Kalamazoo, Michigan, USA, or Enterprise stent, Codman & Shurtleff, Raynham, Massachusetts, USA) with or without coiling or flow diverting device (Pipeline, Medtronic, USA or Tubridge, MicroPort, China). In deploying the flow diverter, it was possible to avoid covering the orifice of the contralateral vertebral artery. For patients with coil embolization, an embolization microcatheter was navigated into the aneurysm cavity for coiling. After embolization, digital subtraction angiography was performed to check the adherence of the flow diverters or stent to the vascular wall, and balloon expansion of the stent was conducted in poor wall adherence. For patients with apparent parent artery stenosis, the stenosis was expanded using a balloon catheter after stent embolization. Procedure success was defined as complete coverage of the aneurysm neck by the stent or flow diverter, good wall adherence, and patent parent artery. Computed tomographic scan was conducted immediately after endovascular treatment so as to exclude possible intracranial hemorrhage.

Clinical follow-up was performed 3, 12 and 24 months after the procedure to check possible adverse events which were defined as cerebral hemorrhage, infarction, and any neurological symptoms. The patients were evaluated with the modified Rankin scale (mRS) score. Angiographic follow-up was conducted with the magnetic resonance imaging angiography or digital subtraction angiography. The aneurysm occlusion status on imaging was assessed with the O'Kelly-Marotta (OKM) grading system (17), with the aneurysm filling grade of A - complete filling (>95%), B - incomplete (5%-95%), C - neck remnant (<5%), or D - no filling (0%).

### Statistical analysis

The statistical analysis was performed with the SPSS 19.0 (IBM, Chicago, IL, USA). Measurement data were presented as mean ± standard deviation if in normal distribution and tested with the *t*-test or median and interquartile range if in skew distribution and tested with the Mann-Whitney U test. Enumeration data were presented as numbers and percentages and tested with the Chi square test. A *P* < 0.05 was considered statistically significant.

## Results

Twenty-five patients with unruptured dissecting intracranial aneurysms of the vertebral artery treated with flow diverters were enrolled as the flow diversion group, including 16 males and 9 females, with an age range of 29–72 (mean 52 ± 11.3) years (Table 1). The symptoms were headache or neck pain in 13 (52%) patients and cerebral infarction or transient ischemic attack in six (24%), and the other six (24%) patients were incidentally found to have dissecting intracranial aneurysms of the vertebral artery. Primary hypertension was found in nine (36%) patients and diabetes mellitus in five (20%). The aneurysm was on the left side in 10 (40%) patients and on the right side in 15 (60%). The aneurysm involved the posterior inferior cerebellar artery in eight (32%) patients, and concomitant stenosis existed in the parent artery proximal or distal to the aneurysm in ten (40%) patients.

**Table 1:** Demography (n, %).

**Variables**		**Flow diversion (*n* = 25)**	**Stent (*n* = 42)**	***P***
F/M		9/16	15/27	0.78
Age (y, range and mean)		29–72 (52 ± 11.3)	32–75 (53 ± 9.3)	0.36
Symptoms	Headache or neck pain	13 (52%)	22 (52.4%)	0.87
	Cerebral infarction or TIA	6 (24%)	13 (31.0%)	0.56
	Incidentally found	6 (24%)	7 (19.0%)	0.23
History	Hypertension (n, %)	9 (36%)	12 (28.6%)	0.54
	Diabetes mellitus	5 (20%)	6 (14.3%)	0.27
Features of aneurysms	Left vertebral artery	10 (40%)	25 (59.5%)	0.56
	Right vertebral artery	15 (60%)	17 (40.5%)	0.21
	Involvement of PICA	8 (32%)	15 (35.7%)	0.66
	Concomitant parent artery stenosis	10 (40%)	12 (28.6%)	0.27

*PICA, Posterior inferior cerebellar artery*.

Forty-two patients with intracranial unruptured dissecting aneurysms of the vertebral artery treated with stent alone or stent-assisted coiling were also enrolled as the stent group, including 15 female and 27 male patients with an age range of 32–75 (mean 53 ± 9.2) years (Table 1). The symptoms were headache or neck pain in 22 (52.4%) patients and cerebral infarction or transient ischemic attack in 13 (31.0%), and the other seven (16.7%) patients were incidentally found. Hypertension was found in twelve (28.6%) patients and diabetes mellitus in six (14.3%). The aneurysm was on the left side in 25 (59.5%) patients and on the right side in 17 (40.5%). The aneurysm involved the posterior inferior cerebellar artery in 15 (35.7%) patients, and concomitant stenosis existed in the parent artery proximal or distal to the aneurysm in 12 (28.6%) patients. No significant (*P* > 0.05) difference was found in the basic information of patients between two groups.

**Figure 1:**
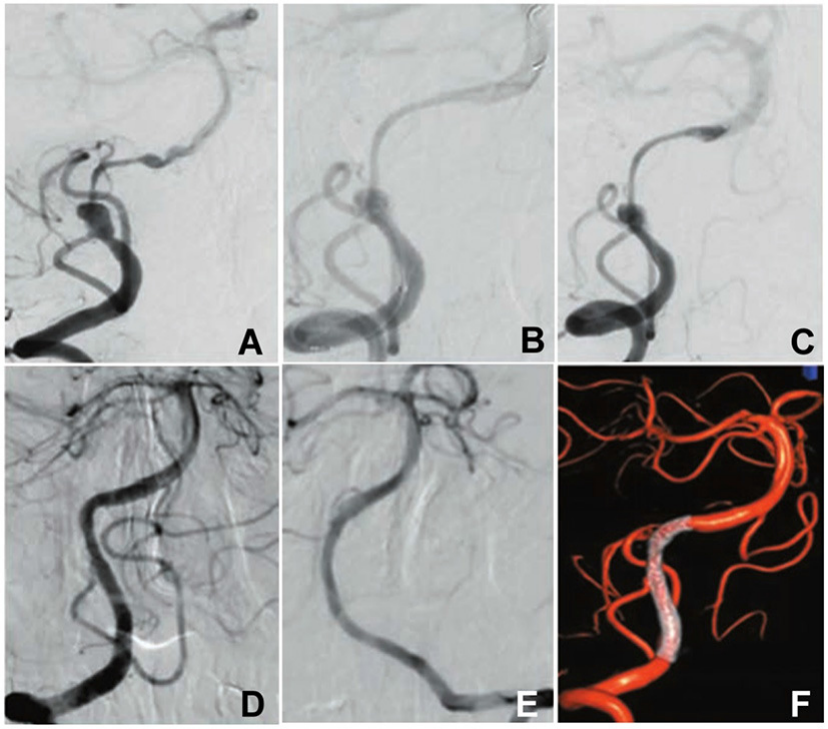
A patient with intermittent headache was hospitalized. **(A)** Cerebral angiography revealed a dissecting aneurysm at the intracranial vertebral artery concomitant with distal long-segment stenosis. **(B,C)** A flow diverter was deployed to cover the aneurysm. **(D)** Angiography 3 months later demonstrated complete occlusion of the dissecting aneurysm with the stenosis being relieved and patent posterior inferior cerebellar artery. **(E)** The left vertebral artery was normal. **(F)** Three-dimensional angiography of reconstruction showed complete occlusion of the dissecting aneurysm and patent flow diverter.

The endovascular stenting procedure was technically successful in all patients receiving the flow diverters, stent alone, or stent-assisted coiling. In the flow diversion group, twenty-six flow diverting devices were deployed in 25 patients, including 16 Pipeline embolization devices (PEDs) deployed in 16 (64%) patients and ten Tubridge devices in nine (36%) patients (Table 2 and Figure 1). One flow diverter was deployed in 24 (96%) patients each, and two Tubridge devices were deployed in one (4%) patient, and additional coiling was performed in two (8%) patients after deployment of one diverter. The diverter deployment technical success was 100%, with complete coverage of the aneurysm neck, good wall adherence, and patent parent artery. Immediate angiography revealed contrast agent retention within the aneurysm cavity in all patients. In ten (40%) patients with moderate parent artery stenosis, no predilatation was needed, and in one (4%) patient with concomitant parent artery stenosis, postdilatation was performed within the stent. In ten (40%) patients, the flow diverter covered the orifice of the posterior inferior cerebellar artery after deployment. No relevant neurological complications Occurred in this cohort of patients.

**Table 2:** Endovascular treatment and follow-up.

**Variables**	**Flow diverter**	**Stent**
Devices deployed (n)	26	48
Technical success rate	100%	100%
Type of device	PED 16 (64%)	NF 28 (58.3%)
	Tubridge 9 (36%)	EP 20 (41.7%)
Treatment mode (*n*, %)		
Patients with 1 device	24 (96%)	36 (85.7%)
Patients with 2 devices	1 (4%)	6 (14.3%)
Additional coiling	2 (8%)	36 (85.7%)
Immediate occlusion outcomes		
OKM grade D	0	18 (42.9%)
OKM grade C	0	16 (38.1%)
OKM grade B	25 (100%)	8 (19.0%)
Clinical follow-up Duration (m)	3–46 (median 22)	6–58 (median 37)
Hemorrhagic complications	1	0
Ischemic complications	0	0
Angiographic follow-up duration (m)	3–46 (median 24)	6–58 (median 34)
No. of patients with follow-up	19 (76%)	33 (78,6%)
DSA	18 (94.7%)	19 (57.6%)
CTA	1 (5.3%)	11 (33.3%)
OKM grade D	11 (57.9%)	22 (66.7%)
OKM grade C	2 (10.5%)	5 (15.2%)
OKM grade B	6 (31.6%)	3 (9.1%)
Recurrence	0	3 (9.1%)
Asymptomatic instent stenosis	2 (10.5%)	5 (16.7%)
Parent artery stenosis improved	5 (71.4%)	6 (20%)

*PED, Pipeline embolization device; DSA, digital subtraction angiography; CTA, computed tomography angiography; OKM grade, O'Kelly-Marotta grading system*.

In the stent group, 48 stents were deployed in 42 patients, including 36 Neuroform (75%) and 20 Enterprise (25%) stents (Table 2). One stent was deployed in each of 36 patients (85.7%) for stent-assisted coiling, and two stents were deployed without additional coiling in each of the other six patients (14.3%). The technical success rate of stenting was 100%. Immediate angiographic outcome showed OKM grade D with no aneurysm filling in 18 (42.9%) aneurysms, OKM grade C with a neck remnant in 16 (38.1%), and OKM grade B with incomplete occlusion in 8 (19.0%). No pre- or postdialation of the stent was performed in these patients. Periprocedural ischemic complications with thrombosis occurred in two (4.8%) patients, and no neurological deficits were left after appropriate thrombolysis.

In the flow diversion group, all 25 (100%) patients underwent clinical follow-up 3–54 months (median 22). In one (4%) patient with sudden headache 8 months after the embolization procedure, hemorrhage of the caudate nucleus was demonstrated on computed tomography to break into the ventricle, and the patient recovered completely after drug treatment. Nineteen (76%) patients underwent angiographic follow-up 3–46 (median 24) months after the procedure, including digital subtraction angiography in 18 (94.7%) patients and computed tomographic angiography in one (5.3%). The OKM grade was D in 11 (57.9%) patients, C in two (10.5%), and B in six (31.6%). No aneurysm recurrence was detected. Asymptomatic instent stenosis occurred in two (10.5%) patients including one patient with deployment of two Tubridge devices. All the other stents remained patent. Seven (70%) of the ten patients with the flow diverter covering the orifice of the posterior inferior cerebellar artery underwent angiographic follow-up, and the posterior inferior cerebellar artery remained patent. In seven of ten patients with mild or moderate parent artery stenosis who experienced angiographic follow-up, the stenosis was improved in five (71.4%) patients.

In the stent group, clinical follow-up was performed 6–58 months after the procedure through clinic visit or telephone contact, with no additional complications. Angiographic follow-up was carried out in 33 (78.6%) patients 6–58 months (median 34) after the procedure, with the OKM grade D in 22 (66.7%) patients, grade C in five (15.2%), grade B in three (9.1%), and recurrence in three (9.1%). Five (16.7%) patients experienced asymptomatic instent stenosis, and six of 12 patients (50%) with parent artery stenosis were improved.

In comparison between the flow diversion and stent groups (Table 2), no significant (P>0.05) differences were found in the number of devices deployed, treatment mode, immediate occlusion outcomes, complications, clinical and angiographic follow-up, follow-up angiographic modes, and follow-up outcomes (OKM grade and stenosis).

## Discussion

In this study investigating the effect and safety of flow diverters in the treatment of unruptured dissecting intracranial aneurysms of vertebral artery in comparison with stent-assisted coiling or stenting alone, it was found that the use of flow diverters for the treatment of unruptured dissecting intracranial aneurysms of vertebral artery was safe and effective with good occlusion effects no less than those of stent-assisted coiling and stenting alone.

Surgical and endovascular techniques can both be used to treat the intracranial dissecting aneurysms of the vertebral artery, however, the surgical approach has been applied less often because the deep location, long segment, fusiform shape, and frequent involvement of the posterior inferior cerebellar artery and brain stem perforators make surgical treatment much more difficult and increase the risk of severe complications (18). Thus, as a microinvasive approach, the endovascular approach has become the primary method of treatment, including arterial reconstruction and occlusion. For unruptured intracranial dissecting aneurysms of vertebral artery, arterial reconstruction is the main treatment approach, including covered stent implantation, stent implantation only, and stent-assisted coil embolization. A covered stent can isolate the aneurysm from the parent artery flow by reconstructing the parent artery, however, the covered stent is less compliant and limited in the stent length and diameter, unsuitable for aneurysms involving perforating arterial branches, longer arterial segment, or larger diameter of the parent artery. Moreover, endoleak, thrombosis, and stent occlusion may occur after deployment of the covered stent, limiting its wide application (19). Stent deployment alone has a certain role in diverting blood flow by partially occluding the intimal tear and limiting blood flow into the aneurysm to promote healing of the dissecting aneurysms, but may not be able to prevent the recurrence and rebleeding (20). Compared with sole stenting, stent-assisted coiling can reconstruct the parent artery, ensure arterial patency, and embolize the aneurysm cavity to decrease the risk of rebleeding, achieving good short-term results even though the primary issue with this technique is still aneurysm recurrence. Because the number of stents deployed correlates with the flow diverting effect and improved hemodynamics, which is beneficial to the prevention of rupture and recurrence of dissecting aneurysms, an increasing number of researchers tend to use multiple stents to assist coiling of dissecting aneurysms (21, 22). The use of multiple stents with or without concomitant coiling may had a similar effect to that of flow diverters. However, the number of stents deployed is also closely related to periprocedural ischemic events, and certain technical difficulties exist in the use of stent-assisted coiling for dissecting aneurysms involving perforating arteries.

The treatment of intracranial aneurysms is changed from intrasaccular embolization to reconstruction of the parent artery by flow diverting devices, which is in line with the treatment idea of multiple stent placement for dissecting aneurysms. Coiling operation within the aneurysm sac is dangerous and may puncture the aneurysm wall, resulting in intra-procedural aneurysm rupture, severe complications or death. Moreover, flow diverters have significantly decreased porosities with an improved effect of flow diversion, reduced blood flow into the aneurysm sac to facilitate thrombosis (23–25), and provided a physical scaffold across the aneurysm neck to promote neointimal growth (23, 26, 27), thus demonstrating a great advantage in the treatment of dissecting aneurysms. Natarajian et al. (28) have reported good outcomes in applying the PED device to treat 12 patients with fusiform vertebrobasilar artery aneurysms sized 13.25 ± 4.5 mm, and at the minimum follow-up duration of 1 year, all 12 aneurysms were occluded, the PED devices were patent, and no patient experienced delayed hemorrhage, stroke, or in-stent stenosis. Zhang et al. (29) have explored the feasibility of PED device compared with stent-assisted coiling in the treatment of non-saccular intracranial vertebral artery aneurysms. They found that the PED device achieved similar procedural complications, angiographic results, and favorable clinical outcomes, with aneurysms treated with PED being more prone to complete occlusion over time than aneurysms treated with the stent-assisted coiling technique, suggesting a safe and feasible strategy of the PED device in the treatment of these aneurysms. In our study, the proportion of dissecting aneurysms obtaining near complete occlusion was 68% (13/19), with no aneurysm recurrence at follow-up. This near complete occlusion rate may be low for some reasons. Firstly, the follow-up time may not be long enough because the complete occlusion degree increases with the follow-up time in flow diversion treatment of cerebral aneurysms. Secondly, most of the dissecting aneurysms were fusiform with a wide aneurysm neck, which may affect the endothelialization of the flow diverter at the neck. Thirdly, fewer cases were treated with overlapped flow diverters, with only two flow diverters deployed in one (4%) patient. Two overlapped flow diverters can increase the metal coverage area and is beneficial to thrombosis within the aneurysm cavity and complete aneurysm occlusion. Adjunctive coiling after deployment of a diverter was performed only in two (8%) patients, and most other patients had deployment of only one flow diverter. Adjunctive coiling will promote aneurysm thrombosis and complete occlusion. The above reasons may account for the low complete occlusion degree in our study.

Because of the rich perforating branches in the basilar artery, more periprocedural complications may occur in the use of flow diverters for the treatment of basilar artery aneurysms (30). However, there are few branches and perforating vessels in the intracranial segment of the vertebral artery, so ischemic complications of flow diverters in the treatment of unruptured intracranial dissecting aneurysms of the vertebral artery are less likely to occur. In our study, no ischemic complications occurred in the periprocedural period or at follow-up. Flow diverters can effectively protect the patency of perforators involved by the aneurysm while completely occluding the aneurysm. In the flow diversion group in our study, among ten patients whose posterior inferior cerebellar artery was covered by the flow diverter, seven patients had cerebral angiographic follow-up which proved patency of the artery. In the stenting group, no ischemic complications occurred at follow-up. In a study by Catapano et al. (5) reporting the endovascular treatment of unruptured vertebral artery dissecting aneurysms (VADAs) and ruptured dominant VADAs using flow diverters and stent-assisted coiling, it was found that the use of flow diverters for the treatment of VADAs seemed to be associated with lower retreatment and complication rates than stenting-assisted coiling (complications 2/29 vs. 4/15, P=0.008 and retreatment 4/29 vs. 6/15, *P* = 0.002) and that endovascular treatment of unruptured VADAs was safe with favorable angiographic and neurological outcomes. This study supports our outcomes.

Compared with the clinoid segment of the internal carotid artery, the intracranial segment of the vertebral artery is relatively straight and smooth, which enables easy deployment of the flow diverter. However, a dissecting aneurysm at this segment is usually fusiform and wide-necked and involves a longer segment, and a longer flow diverting device with a greater diameter than that of the parent artery should be chosen to cover the aneurysm neck with sufficient anchoring area at the distal end. When releasing the device at the aneurysm neck, the delivery micro-catheter should maintain a certain tension, and the device should be mainly pushed to prevent the distal end from sliding downward. For larger aneurysms, loose coil packing is needed to promote the closure of aneurysms.

Intracranial dissecting aneurysms of the vertebral artery may be accompanied by stenosis of the parent artery. In our study, ten (40%) patients exhibited mild and moderate stenosis of the parent artery. Predilatation of moderate stenosis is not necessary because the stenosis will be alleviated after deployment of the flow diverter, and further alleviation of the stenosis will be achieved at follow-up with repair and reconstruction of the parent artery. In seven of these patients who had angiographic follow-up, the stenosis was improved in five (71.4%) patients. In some patients with parent artery stenosis which did not improve after deployment of the flow diverter, postdilatation may be needed to relieve the stenosis as demonstrated in one patient in our study.

Instent stenosis or occlusion is another issue worthy of attention after the placement of a flow diverter. The study by Chalouhi et al. (31) showed that the rate of instent stenosis was as high as 15.8%, but most of the stenoses were asymptomatic. In our study, instent stenosis occurred in two patients, and in one patient with two Tubridge devices deployed in the “bridging form,” the stent was occluded at 3 months after the procedure, which was probably caused by deployment of multiple devices (32).

The involvement of the origin of the posterior inferior cerebellar artery is an independent risk factor affecting recurrence of intracranial vertebrobasilar dissecting aneurysms (33). In our study, patients with the OKM grade B occlusion degree at follow-up had the involvement of the posterior inferior cerebellar artery origin, and long-term effects of these patients remain to be determined. In one patient with hemorrhage of the caudate nucleus to break into the ventricle, the hemorrhage was probably related to poor control of the blood pressure and high blood concentration of antiplatelet therapy.

In the use of flow diverters or intracranial stents to treat cerebral aneurysms, dual antiplatelet therapy is necessary to prevent stent-related thromboembolic events. Recently, an anti-thrombotic polymer coating has been developed for coating a flow diverter (the p48MW HPC), and this coated flow diverter allows application of a single antiplatelet function medication for endovascular treatment of cerebral aneurysms, especially ruptured ones (34–36). Recent exploring studies have proved that deployment of this coated flow diverter is able to decrease thrombogenicity safely and effectively, and achieves good early aneurysm occlusion rates in cerebral aneurysms in the proximal intracranial circulation, complex bifurcation aneurysms in the anterior and posterior circulation, and distally located cerebral aneurysms even though further larger comparative studies are essential to confirm these outcomes and optimize the perioperative antiplatelet treatment (34–36).

This study had some limitations, including the one-center and retrospective design, the small cohort of patients, the enrollment of only Chinese patients, and the lack of randomization, which may all affect the generalization of the outcomes. Further studies will be needed to resolve these issues for better outcomes.

## Conclusion

In conclusion, the use of flow diverters with or without selective coiling for the treatment of unruptured dissecting intracranial aneurysms of vertebral artery may be safe and effective with good occlusion effects not inferior to those of stent-assisted coiling and stenting alone, even though the long-term effect still warrants confirmation.

## Data availability statement

The original contributions presented in the study are included in the article/supplementary material, further inquiries can be directed to the corresponding author.

## Ethics statement

The studies involving human participants were reviewed and approved by Ethics Committee of Henan Provincial People's Hospital. The patients/participants provided their written informed consent to participate in this study.

## Author contributions

B-LG and T-XL: study design. LL, H-LG, G-QX, KZ, and Z-LW: data collection. G-QX, B-LG, and T-XL: data analysis. LL: writing of the original version. B-LG: revision. All authors: validation and approval.

## Funding

This study was supported by the 13th Five-year Plan of China for Research and Development (2016YFC1300702), Henan Province Science and Technology Key Project (182102310658), and Scientific and Technological Project of Henan Province (222102310208).

## Conflict of interest

The authors declare that the research was conducted in the absence of any commercial or financial relationships that could be construed as a potential conflict of interest.

## Publisher's note

All claims expressed in this article are solely those of the authors and do not necessarily represent those of their affiliated organizations, or those of the publisher, the editors and the reviewers. Any product that may be evaluated in this article, or claim that may be made by its manufacturer, is not guaranteed or endorsed by the publisher.
